# Reflections on the Financial Toxicity of Cancer: 10 Years Later

**DOI:** 10.1093/oncolo/oyad179

**Published:** 2023-06-14

**Authors:** S Yousuf Zafar, Jeffrey Peppercorn

**Affiliations:** Department of Medicine, Duke University School of Medicine, Durham, NC, USA; Department of Medicine, Massachusetts General Hospital Cancer Center, Boston, MA, USA

## Abstract

This commentary reflects on the decade since the publication of an article on the financial toxicity of cancer treatment and the international recognition of the scope and depth of the problem, in the hope that the coming decade is characterized by evaluation and demonstration of optimal solutions to the problem.

In 2008, the authors of this article were still early in their careers and practiced at the same institution. In the midst of the Great Recession, patients were concerned not only about the care they would receive, but about their personal financial responsibility for cancer care. Patients would ask for less expensive prescription medications, or they would ask to complete CT scans and other procedures before their insurance deductible renewed in the new year. Prior to the 2010 Affordable Care Act, close to 44 million Americans (15% of the population) lacked health insurance, and costs of health care were a rising concern even for patients with commercial insurance plans.^1^ In 2008, amid the economic downturn, 28% of Americans reported serious problems paying for their health care or health insurance. A year later, as health care reform was again debated in Washington and as cancer drug prices continued to rise, the American Society of Clinical Oncology (ASCO) published its landmark guidance statement on the cost of cancer care.^2^ The article appropriately took a multi-stakeholder approach and focused on the factors contributing to the high societal costs of care. In addition, the article acknowledged that the financial burden of care was being shifted to patients in the form of increasing out-of-pocket costs, and as a result, ASCO recommended prioritizing patient-physician discussions on treatment affordability.

In the same year that the ASCO guidance was published, the authors of this commentary (S.Y.Z., J.P.) came across a career development award in access to care from the HealthWell Foundation, a non-profit that provides financial assistance for health care-related out-of-pocket expenses. Considering what the authors had seen in the growing literature on the topic, and in light of their own patients’ experiences, the authors applied for and were awarded a grant entitled, “Out-of-pocket expenses and access to cancer care.” That grant ultimately led to a manuscript, “The Financial Toxicity of Cancer Treatment: A Pilot Study Assessing Out‐of‐Pocket Expenses and the Insured Cancer Patient’s Experience,” published a decade ago in *The Oncologist*.^3^ This article was by no means the first to address the influence of cost on the cancer patient experience; indeed, it was not even the first published in this journal.^4,5^ However, it has garnered considerable attention over the years. One possible reason for the lasting influence of this work is the term, “financial toxicity.” According to PubMed, that manuscript was the first to mention the term in the published academic literature. Since then, over 700 PubMed-indexed articles have mentioned “financial toxicity,” with Google News listing thousands of articles—the most recent published just days before this writing. Indeed, the term financial toxicity has now “metastasized” well beyond oncology.^6,7^

Why the lasting impact? Anyone touched by cancer—from clinician, to patient, to caregiver—intuitively understands the concept of toxicity resulting from cancer treatment. The language of physical harm as a potential side effect from therapy is familiar and easy to translate for a lay audience. The language of health economics can be harder to grasp. “Financial toxicity” aligned the sometimes invisible cost burden patients and families experience with the well-understood and palpable physical toxicity of treatment, thereby capturing the imagination of clinicians, patients, the lay media, and other industry stakeholders including the pharmaceutical industry. But a term that draws attention in the press can oversimplify a multifaceted concept. One term cannot adequately describe how cost might influence a patient’s experience.^8^ For example, financial burden might describe the objective costs faced by a patient, but even with relatively low absolute costs, a patient or family might still experience subjective financial distress. Additionally, “financial toxicity” can sometimes conflate the challenge of out-of-pocket financial burden for an individual with the unsustainable societal costs of care, a related but distinct issue.^9^ Indeed, considerable research has been conducted on how to best measure the objective and subjective harm from costs, from single questions to validated multi-item surveys.^10^ Nonetheless, the term has to some degree helped to place the issue of financial burden before researchers, research funding organization, and journals.

The field of research focused on patients’ experience with financial burden has blossomed in the past decade. Much of the initial and ongoing work has been descriptive in nature and has demonstrated a considerable bearing on patient well-being and care. The literature suggests that patient financial toxicity is common and seen in all cancer settings, often regardless of care modality.^10^ However, certain patient populations are at greater risk of experiencing financial toxicity, including younger, nonwhite, female, and low-income patients.^11^ Importantly, financial toxicity often extends into survivorship after active treatment has ended. Multiple studies have demonstrated an association between financial toxicity and emotional distress, quality of life, and even physical symptom burden.^10^ The reach of financial toxicity extends into quality of care, with a correlation between high financial toxicity and access to care, adherence to treatment, and enrollment in clinical trials.^12^ Furthermore, it can play a varying role in patient decision-making and the patient-clinician relationship. Most patients want to know their costs in advance.^13,14^ Typically, these patients are not only willing to discuss costs, but also seek help mitigating financial burdens. A minority of patients do not want to discuss costs, so individualized attention to patient decision-making preference is appropriate.^14^ Studies have also investigated who on the care team should bring up the topic of costs with patients and family members and whether the physician is the best team member to initiate that discussion.^15^ Most recently, a growing number of trials are testing novel interventions to reduce financial toxicity through price transparency, better communication, or financial navigation.^16,17^

Research on financial toxicity naturally lends itself to a policy focus, and some key opportunities have arisen over the past decade to examine health policy through the lens of patient financial burden. The most impactful, of course, been the passage of the Affordable Care Act (ACA). Without question, the ACA has improved affordability of health care *coverage*, with the rates of uninsured Americans reduced by nearly half compared to the pre-ACA era.^18^ However, the ACA arguably did little to reduce affordability of health care through lower out-of-pocket health care spending. Annual maximums on out-of-pocket spending have been offset by rising premiums and deductibles, such that in 2022, a record-high proportion of Americans delayed medical treatment due to cost.^19^ Another landmark health policy that aims to reduce health care costs is the Inflation Reduction Act (IRA), signed in 2022. The greatest potential for patient cost reductions will likely manifest through caps on out-of-pocket spending for Medicare Part D enrollees.^20^ Yet the components of the IRA garnering the most attention are the mandates on drug manufacturers to negotiate certain drug prices with the federal government. We are unlikely to see near term reductions in out-of-pocket costs as a result of price negotiations, and the pharmaceutical industry is already preparing for these negotiations by raising drug prices for 2023.^21^

In the past decade, a tremendous body of work has detailed the repercussions of financial toxicity on the patient experience. Where do we go from here? We must acknowledge that solutions to rising health care costs are challenging and require multi-stakeholder engagement to ensure affordable, sustainable high-quality care. At the same time, we must recognize that financial toxicity is a byproduct of misaligned incentives among those stakeholders. Drug manufacturers are incentivized to bring higher-priced “me-too” drugs to market rather than innovate.^22^ Insurers are incentivized to reduce care utilization rather than improve coverage. Further, health systems and many clinicians are incentivized to provide costly interventions rather than promote healthy preventative behavior. This problem of misaligned incentives is not unique to the U.S., but it is exacerbated by the predominance of our fee-for-service care delivery system.

One potential path to more affordable health care is value-based care, where payment and coverage is based not on volume, but on the quality of care delivered. Alongside value-based drug pricing and approval, value-based care delivery could align incentives by promoting medical innovation, evidence-based insurance coverage, and quality-focused—rather than volume-focused—care delivery. Importantly, insurance coverage would have to be restructured to minimize or eliminate out of pocket costs for high value care if this approach is to save money for both the payer and the patient. Yet, while we have been discussing value-based care for decades, its implementation remains in its relative infancy. Policy research must investigate means to expand effective value-based care delivery beyond our current state. At the patient and provider level, funding agencies should now prioritize randomized trials that test the impact of navigation, price transparency, and other cost-reducing interventions. Most importantly, research on financial toxicity must continue to amplify the voice of the patient and caregiver and their experiences with high costs of care.

If the decade since the publication of our 2013 paper on financial toxicity in the pages of *The Oncologist* was characterized by a truly international recognition of the scope and depth of the problem, our hope is that the coming decade is characterized by evaluation and demonstration of optimal solutions, so that the distress and toxicity conveyed by a cancer diagnosis, is not compounded by financial burdens and barriers, and that all patients have secure access to highest quality cancer care.
